# Uptake and subcellular distribution of radiolabeled polymersomes for radiotherapy

**DOI:** 10.7150/ntno.37080

**Published:** 2020-01-01

**Authors:** Stefan J. Roobol, Thomas A. Hartjes, Johan A. Slotman, Robin M. de Kruijff, Guzman Torrelo, Tsion E. Abraham, Frank Bruchertseifer, Alfred Morgenstern, Roland Kanaar, Dik C. van Gent, Adriaan B. Houtsmuller, Antonia G. Denkova, Martin E. van Royen, Jeroen Essers

**Affiliations:** 1 Department of Molecular Genetics, Erasmus University Medical Center, Rotterdam, The Netherlands; 2 Oncode Institute, Erasmus University Medical Center, Rotterdam, The Netherlands; 3 Department of Radiology & Nuclear Medicine, Erasmus University Medical Center, Rotterdam, The Netherlands; 4 Department of Pathology, Erasmus University Medical Center, Rotterdam, The Netherlands; 5 Optical Imaging Centre (OIC), Erasmus University Medical Center, Rotterdam, The Netherlands; 6 Department of Radiation Science and Technology, Delft University of Technology, Delft, The Netherlands; 7 European Commission, Joint Research Centre, Directorate for Nuclear Safety and Security, Karlsruhe, Germany; 8 Cancer Treatment Screening Facility (CTSF), Erasmus University Medical Center, Rotterdam, The Netherlands; 9 Department of Radiation Oncology, Erasmus University Medical Center, Rotterdam, The Netherlands; 10 Department of Vascular Surgery, Erasmus University Medical Center, Rotterdam, The Netherlands

**Keywords:** Polymersomes, uptake, radionuclide therapy, live cell confocal microscopy, nano-carriers

## Abstract

Polymersomes have the potential to be applied in targeted alpha radionuclide therapy, while in addition preventing release of recoiling daughter isotopes. In this study, we investigated the cellular uptake, post uptake processing and intracellular localization of polymersomes. **Methods:** High-content microscopy was used to validate polymersome uptake kinetics. Confocal (live cell) microscopy was used to elucidate the uptake mechanism and DNA damage induction. Intracellular distribution of polymersomes in 3-D was determined using super-resolution microscopy. **Results:** We found that altering polymersome size and concentration affects the initial uptake and overall uptake capacity; uptake efficiency and eventual plateau levels varied between cell lines; and mitotic cells show increased uptake. Intracellular polymersomes were transported along microtubules in a fast and dynamic manner. Endocytic uptake of polymersomes was evidenced through co-localization with endocytic pathway components. Finally, we show the intracellular distribution of polymersomes in 3-D and DNA damage inducing capabilities of ^213^Bi labeled polymersomes. **Conclusion:** Polymersome size and concentration affect the uptake efficiency, which also varies for different cell types. In addition, we present advanced assays to investigate uptake characteristics in detail, a necessity for optimization of nano-carriers. Moreover, by elucidating the uptake mechanism, as well as uptake extent and geometrical distribution of radiolabeled polymersomes we provide insight on how to improve polymersome design.

## Introduction

Targeted alpha therapy (TAT) is considered as treatment option for various tumours, such as bladder cancer, brain tumours, neuroendocrine tumours, and prostate cancer 1, 2. Alpha particles have high linear energy transfer (LET) and therefore result in a higher relative biological effect (RBE), compared to low-LET radiation. The range of alpha particles is up to 100 μm in water, while it is even more limited in tissue 3. These distances only span a few cells, thereby limiting damage to surrounding healthy tissue 4. In current experimental therapy settings, short-lived radionuclides require rapid targeting for efficient dose delivery to target cells 5. Long-lived radionuclides can overcome these limitations, but are often part of a longer decay chain which leads to the release of recoiling daughter isotopes. Recoiling daughter isotopes break free from their targeting vehicle, such as antibodies and peptides, and can distribute freely in the body, potentially causing harm to healthy tissue 6, 7. This problem can be solved by the use of nano-carriers, e.g. liposomes. While the retention of the mother nuclides in liposomes is up to 98%, the retention of the recoiled daughter isotopes is less than 20% 8. Low retention makes liposomes poor carriers of high-LET radionuclides with multiple alpha emitting isotopes in the decay chain. Nano-carriers composed of polymers, such as dendrimers, polymeric particles, nano-gels and micelles are more robust and are thereby more effective in retaining the daughter isotopes compared to other nano-carriers 9-11.

Polymersomes (PMs), formed through self-assembly of amphiphilic block copolymers, combine the possibility to counter the recoil problem and have highly versatile adjustable properties, making them attractive candidates for customized high-LET radionuclide 12, 13. Nano-carriers are commonly known to be delivered at the tumor site via the enhanced permeability and retention (EPR) effect 14, 15. This effect is mostly observed in rapidly growing solid tumors and their high demand for oxygen and nutrients. This high demand causes underdeveloped and leaky vasculature in and around the tumor. PM therapy can exploit this phenomenon, which allows passive PM transfer to tumor areas via the blood circulation. Variation in size could provide beneficial effects on circulation times and mechanical filtration 16. Recent reports show that variations in charge and degree of attached polyethylene glycol (PEG) affect PM uptake, circulation time and clearance pathways 12, 16-19. In addition, intra-tumoral injections show high retention in tumor tissue, which could indicate intracellular uptake of PMs and not accumulation in the extracellular matrix 19, 20.

Although the uptake of other nano-carriers in cells has been documented, to our knowledge, no reports show the cellular and biological uptake mechanism of PMs. Recently, several reports show the use of PMs for TAT in both *in vitro* and *in vivo* experiments, suggesting that PMs can be used in a therapeutic setting 21, 22. The short range and high-LET of alpha particles requires prolonged localization close to the target cells, which can be reached if PMs are geographically fixed by cellular uptake. A better understanding of the precise uptake mechanism and geometrical distribution of the PMs is crucial to understand how they exert their cell-killing effect in different cell populations. With the use of high-content, confocal (live cell) and super-resolution imaging we evaluate cellular uptake kinetics and post-uptake processing of PMs.

## Materials & Methods

### Polymersome preparation and characterization

PMs with average diameters of 60 and 80 nm were prepared according to the 'inverse nanoprecipitation method' 23. In short, the amphiphilic diblock copolymers (polybutadiene-d-polyethyleneoxide (PBd_1800_PEO_900_)) were dissolved in 1 mL acetone in a 4 ml glass vial (Rotilabo®), using a Vortex-Genie 2 (Scientific industries, Inc.) to obtain a 20 mg/mL block copolymer concentration. The solution was filtered using a 0.20 µm syringe filter (PFTE, unsterile, Rotilabo®). Afterwards, 50 vol % PBS was added using an Aladdin programmable syringe pump (World Precision Instruments, LLC) and a 2 mL Injekt™ syringe (B Braun) under magnetic stirring on a Standard Stirrer (VWR®) at 300 rpm. The remaining acetone was evaporated using a Rotary Evaporation at 30 degrees for at least 15 minutes. Samples of size 400 nm were prepared according to the 'direct dissolution method' 24. In short, 10 mg/mL block copolymer was added to a 1 mM DTPA PBS solution at pH 7.4, and stirred for a week. Subsequently, the PMs were extruded to the required diameter by passing them several times through polycarbonate filters with cut-off membrane of 400 nm. PMs used for radiolabeling were passed through a 30 cm x 0.5 cm (L x r) Sephadex G 25 M size exclusion column (Sigma-Aldrich) to remove excess DTPA.

The size and shape of the PMs were determined by Dynamic Light Scattering (DLS) and Cryogenic-Transmission Electron Microscopy (Cryo-TEM). The DLS apparatus consisted of a JDS Uniphase 633 nm 35 mW lasers, an ALV sp 125 s/w 93 goniometer, a fiber detector and a photon counter (Perkin Elmer). An ALV-500/epp correlator was used to obtain the size correlation function. Scattering cells of 3 mL with an internal diameter of 12 mm were immersed in a temperature regulated toluene bath. The intensity auto-correlation function was determined at 90 degrees. The autocorrelation function was analyzed by the Contin method 10 and the radius of the PMs was determined using Einstein-Stokes equation.

Cryo-TEM characterization was done as described before 11. In short, 3 μL of a 10 mg/mL PMs solution was deposited on a holey carbon film (Quantifoil 2/2) supported on a TEM grid. The sample was blotted and vitrified by rapid immersion in liquid ethane (Leica EM GP version 16222032), and subsequently immersed in liquid nitrogen. A cryo-transfer holder (Gatan model 626) was used to transfer to a Jeol JEM 1400 TEM and images were acquired at an acceleration voltage of 120 keV. For diameter determination, 30-50 images were made of PM samples and measured using FIJI 25.

### Fluorescent labeling and Quantification

Membrane labeling of PMs was done using a fluorescent moiety attached to a lipophilic tail with optimal excitation at 551 nm and emission at 567 nm (PKH26, Sigma-Aldrich) or with optimal excitation wavelength at 490 nm and emission at 502 nm (PKH67, Sigma-Aldrich) according to manufacturer protocol. In short, 20 μL of PMs (10 mg/mL) and 5 μL PKH-dye (working concentration of 2.5E-5 M) were separately diluted in provided Diluent C to 100 μL end volume. Hereafter, the two solutions were mixed and incubated for 10 minutes. After the 10-minute incubation step the PMs are separated from unbound PKH dye using an Exosome Spin Column (Sigma-Aldrich) according to manufacturer protocol. Columns allow buffer exchange on the PMs or to remove any low molecular weight (MW ≤ 3000) mixtures from the preparation. In short, provided spinning columns were solidified using 650 μL PBS and incubated for 10 minutes. Excess PBS was removed by centrifuging the column for 2 minutes at 750 x g. 100 μL of labeled PM solution was then added to the column and centrifuged for 2 minutes at 750 x g, leaving only labeled PMs in Diluent C solution. PKH labeled PMs were quantified by an newly developed confocal fluorescent microscopy assay (EVQuant) 26. In short, fluorescently labeled particles were immobilized in a transparent gel and imaged using an Opera Phenix High Content Screening (HCS) System (Perkin Elmer) and analyzed using Harmony 5.4 (Perkin Elmer). Absolute concentration of in-gel immobilized particles is derived from the number of detected fluorescent particles in the calibrated volume of the imaged region.

### Radioactive labeling

Elution of ^213^Bi was performed as described before 27. The elution mixture was composed of 0.3 mL, 0.2 M HCl and 0.3 mL 0.2 M NaI and pumped through the generator at a flowrate of 0.15 mL/min into a vial containing 0.12 mL of 4 M sodium acetate buffer.

For PM labeling, 700 μL of ^213^Bi was added to a mixture of 10 μL 20 mM tropolone and 100 μL 100 mM Hepes. This solution was incubated for 15 min at RT allowing the ^213^Bi to bind with tropolone. Subsequently, 200 μL 1 mM DTPA encapsulated PMs with a concentration of 1E13 fluorescently labeled PMs/mL was added to the mixture and incubated for 1 hour. Next, the activity was measured by using a NaI detector before and after the column purification of the solution to calculate the loading efficiency of the PMs. Column purification was done using a Sephadex G 25 M column (Sigma-Aldrich) to remove excess DTPA. ^213^Bi retention was similar to previously reported labeling methods for ^111^In and ^225^Ac 10. 100 μL 10 mM DTPA was added to 0.5 mL polymersomes loaded with ^213^Bi and equilibrated for 15 minutes at room temperature. Subsequently, the solution was passed through a Sephadex PD10 column to separate the Bi-DTPA complexes from the polymersomes. The elution was portioned per mL and the ^213^Bi activity in each eluted fraction was determined by dividing the activity detected in the polymersomes by the total activity before separation. At the dose used, no difference in size and physical characteristics was expected 28.

### Cell Culture

U2OS (Human Bone Osteosarcoma), J774A.1 (Mouse Balb/c Monocyte Macrophage) and CA20948 (Rat Pancreatic Cancer) cell lines were cultured in Dulbecco's modified Eagle's medium supplemented with 1% Penicillin/Streptomycin and 10% Fetal Calf Serum. The C5Ro (Human fibroblast) cell line was cultured in Hams' F10 culture medium supplemented with 1% Penicillin/Streptomycin and 15% Fetal Calf Serum. DU145 (Prostate Cancer, CNS Metastasis) and PNT2C2 (Prostate Epithelial) cell lines were cultured in RPMI 1640 medium supplemented with 1% Penicillin/Streptomycin and 5 or 10% Fetal Calf Serum. Mouse embryonic fibroblasts (MEFs) were cultured in 50% Dulbecco's modified Eagle's medium and 50% Hams' F10 medium supplemented with 1% Penicillin/Streptomycin and 10% Fetal Calf Serum. All cells were incubated at 37 ºC in a water saturated atmosphere with 5% CO_2_.

### High-content microscopy

To visualize and quantify PM uptake, cells were seeded (10.000-20.000 cells per well) in 96-wells plates (Sensoplate, Greiner Bio) in duplicates and cultured for 24h. Cells were incubated with PKH26-labeled PMs of different sizes (60, 80 and 400 nm in diameter) and different concentrations (1E10, 2E10, 5E10 or 1E11 PMs/mL) for 1, 2 and 3 hours prior to fixation (4% PFA, 30 min). Cells were washed with PBS and plasma membrane was labeled using PKH67 (Sigma-Aldrich) according to the manufacturers protocol. In short, fixed cells were incubated in 50 μL Diluent C containing PKH67 (2 μL/mL). Samples were stored in 200 μL PBS containing Hoechst (1:10000) for nuclear staining. Fluorescent images were acquired using an Opera Phenix HCS system (Perkin Elmer). For each well, 25 Images were acquired using a 20x objective (NA = 0.4) and the appropriate laser lines and emission filters (Hoechst; ex. 405 nm - em. 435-480 nm, PKH67; ex. 488 nm - em. 500-550 nm and PKH26; ex. 561 nm - em. 570-630 nm. Images were analyzed by the Opera Phenix analysis software (Harmony 5.4) to quantify the number of PMs per cell.

### Confocal (live cell) microscopy

To capture dynamic events of PM uptake and processing, cellular uptake of PMs was analyzed by high-speed Spinning disk microscopy (Nikon Ti-Eclipse and ROPER FRAP3D unit). To study PM uptake in living cells, we used a PNT2C2 cell line stably expressing CAAX-GFP 29. For microtubule imaging PNT2C2 cells were transiently transfected using Tubulin-YFP (YFP-β-tubulin expression construct was kindly provided by Dr. Galjart, Erasmus University Medical Center). For PM uptake experiments, 1E11 PKH26-labeled PMs/mL were added to cells and image acquisition was started using intervals of 500/1000 ms. PM movement speed was analyzed using the TrackMate plugin in FIJI 30.

For co-localization experiments PNT2C2 cells were transiently transfected with Rab4a-YFP (early-endosomes, 31) or incubated with LysoTracker Red (lysosomes, Invitrogen). Rab4a-YFP transfected cells were incubated with PKH26-labeled PMs and LysoTracker Red labeled cells were incubated with PKH67-labeled PMs. Cells were incubated with 1E11 PMs/mL for 30 minutes, washed with PBS and fixed at time points 30, 60, 90, 120, 180, 240, 300 and 360 minutes after starting PM incubation. Fluorescent laser scanning confocal microscopy (CLSM510, Zeiss) was used to capture at least 10 cells per time point for both endocytic markers. Co-localization analysis was performed using FIJI and a customized FIJI macro. In short, in every image both PMs and endocytic compartments in transfected cells were identified and masked based on thresholding the two fluorescent channels. Subsequently, the number of PMs overlapping with endocytic compartments was divided by the total number of PMs to calculate the percentage of co-localization for each image.

To visualize the DNA-damage induced by radioactive loaded PMs, U2OS cells were transiently transfected with 53BP1-GFP (marker for DNA double-strand breaks, full length 53BP1) 32. Transfected cells were treated with ^213^Bi- and PKH26 labeled PMs. ^213^Bi was used as damaging agent and PKH26 was used for visualization of PMs. Cells were treated for 3 hours, washed with PBS and fixed. Confocal microscopy (Leica SP5) was used to capture cells with and without PMs. The numbers of DNA damage clusters were manually quantified. To investigate the fate of 53BP1 clusters induced by alpha particle irradiation, U2OS cells were transiently transfected with mScarlet-53BP1. Truncated 53BP1 (t53BP1, 33) was fused to mScarlet and inserted into a homemade PiggyBac construct. Plasmid and construct information is available on request. Transfected cells were externally irradiated using previously described methods 34. Live cell imaging was performed on a confocal microscope (Leica SP5) overnight (16 hours). Number of cells going through mitosis was manually quantified.

### Super resolution imaging and distance distribution analysis

For intracellular distribution analysis of intracellular PMs, structured illumination microscopy (SIM) was used. PNT2C2 cells were incubated with 1E10 PMs/mL for 2 hours. SIM imaging was performed on a Zeiss Elyra PS1 with an Andor iXon DU 885 EMCCD camera (Carl Zeiss AG, Oberkochen, Germany) using 488, 561 and 642 nm laser excitation with 100 ms exposure times. Samples were illuminated with a spatial line pattern that was shifted in five phases and rotated in five orientations. The raw images were reconstructed into a high‐resolution 3D‐dataset using the Zeiss 2012 PS1 ZEN software. Reconstruction was done using default settings.

The location of each PM in an image was determined using the find maxima algorithm in FIJI. Distribution of PM distance to the nucleus was measured for each cell using intensity profiles of the lines between the center of the nucleus and all PMs. The distance was defined as the length from the PM up to the onset of the nuclear staining. Cutoff distance was determined according to the decay chain of ^213^Bi to ^209^Pb, mainly emitting an alpha particle of 8 MeV 35.

## Results

### Kinetics of polymersome uptake in different cell-types

**Figure 1:**
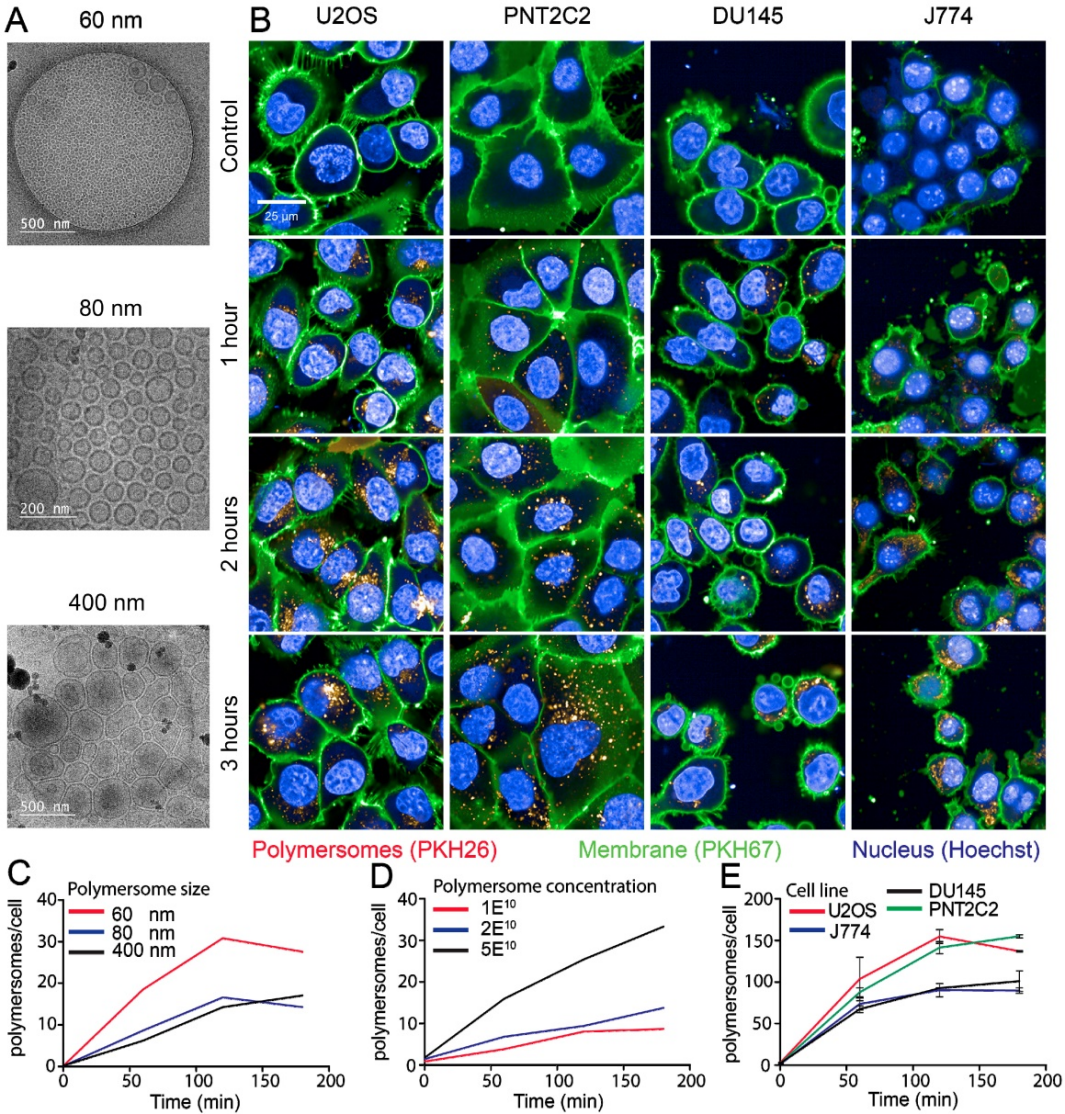
** Uptake kinetics of polymersomes. (A)** Cryo-TEM analysis of PMs dissolved in water **(B)** U2OS, PNT2C2, DU145 and J774 cells labeled with PKH67 (green) as membrane marker were incubated with 80 (±11) nm PMs labeled with PKH26 (red). Cells were fixed at indicated time points and imaged using a high-throughput Opera Phenix system. **(C)** Quantitative analysis of 60 (±8) nm (red), 80 (±11) nm (blue) and 400 (±7) nm (black) sized PM uptake in U2OS cells. **(D)** Uptake analysis of 80 (±11) nm sized PMs in PNT2C2 cells at 1 (red), 2 (blue) or 5 (black) times the original concentration used for uptake experiments. (E) Quantitative analysis of uptake in U2OS (Red), J774 (blue), PNT2C2 (green) and DU145 (black) cells incubated with 80 (±11) nm sized PMs. Error bars indicate SEM, technical replicates. N>1000 cells per condition.

**Figure 2:**
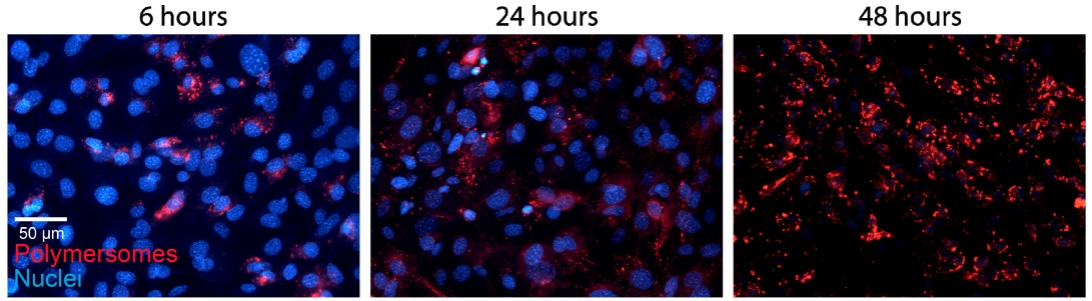
** Polymersome uptake.** MEFs were incubated with 80 (±11) nm sized PKH26 labeled PMs. Cells were fixed at indicated time points and imaged using a wide field epifluorescent microscope (Axio Imager D2, Zeiss).

The PMs used in our experiments are composed of polybutadiene (PBd) and polyethylene oxide (PEO) block co-polymers and were formed in solution by 'inverse nanoprecipitation' (for PMs <100 nm) or 'direct dissolution' (for PMs >100 nm). PMs were characterized by Cryo-TEM and DLS, which showed that the obtained PMs had size distributions of 60 (±8), 80 (±11) and 400(±7) nm in diameter (Figure 1A). To investigate the cellular uptake mechanism of PMs we used different cell types, U2OS (bone osteosarcoma), PNT2C2 (prostate epithelial), DU145 (prostate cancer) and J774A.1 (mouse macrophage, from now on referred to as J774). Exploiting the bi-layered membrane of PMs we employed PKH-dyes for *in vitro* fluorescent tracking. PKH-dyes are composed of intense fluorescent moieties attached to long lipophilic tails. Diffusion of the lipophilic tails in the bi-layer, leaves the fluorescent moiety exposed for PM tracking. Using both PKH26 (Excitation 551 nm/Emission 567 nm) and PKH67 (Excitation 490/Emission 502) we labeled PMs suitable for multiple color combinations. The concentration of detected PMs was measured by the recently developed nanoparticle quantification assay EVQuant 26. The EVQuant assay revealed a labeling efficiency of up to 80% for PKH dyes and concentrations of up to 7.64E13 PMs/mL. Pilot experiments in mouse embryonic fibroblasts (MEFs) showed clustered PM influx using wide-field microscopy, from 6 hours post incubation onwards to 48 hours (Figure 2). Due to this observation we chose for earlier time points and confocal microscopy in the uptake assays for optimal quantification. For uptake experiments the cells were incubated with PMs (80±11 nm) and fixed at 1, 2 or 3 hours post incubation. No background signal at control conditions was observed and PM uptake did not affect cell morphology. Over the course of 3 hours, PMs gradually entered the intracellular compartments of all cell types (Figure 1B). Most notably, the distribution of PMs at 3 hours showed a perinuclear positioning in U2OS cells compared to a random distribution in PNT2C2 cells.

To determine the influence of PM size and concentration on the uptake kinetics in different cell types we employed high-content microscopy for quantitative measurements. As differences in nano-particle size could influence cellular uptake kinetics, we first compared uptake rates of 60, 80 and 400 nm diameter PMs in U2OS cells. In a 96-well plate, 10,000 U2OS cells per well were incubated with 2E10 PKH26-labeled PMs/mL for 1, 2 and 3 hours. Cells were washed with PBS and the cell membranes and nuclei were stained by PKH67 and Hoechst, respectively. Faster uptake kinetics were observed for PMs with 60 nm in diameter compared to 80 and 400 nm. Cells incubated with 60 nm PMs reached a higher plateau level at 3 hours post addition compared to both 80 and 400 nm sized PMs (Figure 1C). Next, various concentrations of initial stock concentrations of PMs were assessed for uptake using PNT2C2 cells using the same high-content microscopy set-up. Cells were incubated for 15, 30, 60, 90, 120 and 180 minutes with 1E10 PMs/mL, 2E10 PMs/mL, and 5E10 PMs/mL of 80 nm diameter PMs. PM uptake is linear with the concentration we used (an average of 2.5 times more PMs at 120 min, between 2E10 and 5E10 PMs/mL, Figure 1D).

Finally, to assess possible differences among cell lines, four different cell lines were incubated with 1E11 PMs/mL. Comparison between different cell lines revealed similar uptake kinetics over time for each of the four cell lines reaching a plateau after 2 hours, possible caused by cellular restrictions of further uptake. Interestingly, the U2OS and PNT2C2 cell lines and the J774 and DU145 cell lines showed a difference in plateau levels (Figure 1E). Altogether, we found that uptake of PM was 2-fold higher for 60 nm PMs compared to 80 nm and 400 nm PMs. Moreover, uptake increased linearly from 1E10 to 5E10 PMs/mL. Finally, the maximum number of PM taken up differed among cell lines.

### Uptake and dynamic processing of polymersomes

In previous high-content screens we observed that PM uptake already occurs in the first 15 minutes after addition. We therefore wanted to capture the immediate response of cells after addition of PMs. To visualize the cellular membrane and transport across this membrane we used a PNT2C2 cell line which stably expresses CAAX-GFP. The CAAX-motif is a target for prenylation 36, and when attached to the GFP protein, it will target GFP to the plasma membrane. CAAX-GFP is therefore a fluorescent marker of the cell membrane, which was used for live-cell imaging of PMs entering the cytoplasmic compartment of cells. Imaging with intervals of 20 min was started immediately after additions of PMs. Interestingly, cells undergoing mitosis (the two upper cells) showed dramatically higher uptake of PMs than cells in other phases of the cell cycle (lower cells, movie S1, scale bar: 20 µm).

**Figure 3:**
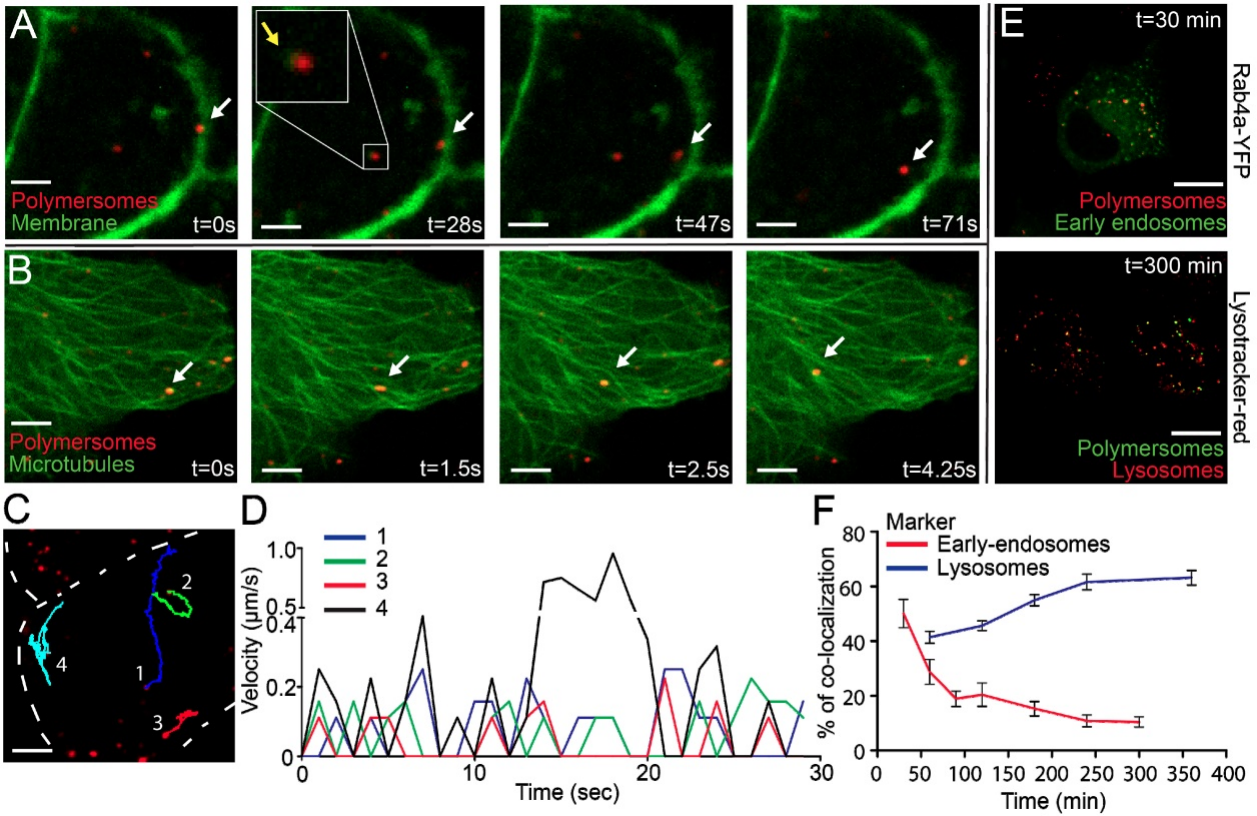
** Rapid uptake, microtubule processing and co-localization of the endocytic pathway of polymersomes.** PNT2C2 cells stably expressing either CAAX-GFP or transiently expressing Tubulin-YFP were incubated with 80 (±11) nm sized PMs (PKH26 labeled, red) and imaged using Spinning Disk Confocal Microscopy. **(A)** Stills of PM uptake, showing attachment (0s), uptake (28-47s) and intracellular processing (47-71s) of PMs, indicated by white arrows. CAAX-GFP component (in green) encircles PMs inside the cell, indicated by yellow arrows. Scale bar represents 5 µm. **(B)** Stills of PM processing. Microtubule labeled by Tubulin-YFP, green. Arrows indicate PMs moving along microtubules. T=0 represents the start of imaging. Scale bar represents 5 µm. **(C)** Representative image of 4 PMs which were tracked over-time. Cell membrane is represented by the dashed line. Scale bar represents 5 µm. **(D)** Velocity of 4 PMs over 30 seconds in a cell. PMs were tracked using Manual Tracking in FIJI. **(E)** DU145 cells (expressing Rab4a-YFP or incubated with Lysotracker-Red) were incubated with 80 (±11) nm PMs (containing PKH26 or 67) and fixed at various time points. Rab4a co-localization and Lysotracker co-localization at 30 and 300 min. Scale bar represents 25 µm. **(F)** Quantification of PM co-localization with early endosomes and lysosomes in time. Error bars indicate SEM, N=10 cells per time point.

To shorten the time interval, we used high-speed spinning disk microscopy which allowed recording of events with minimal time intervals as short as 500 ms. Captured movies are represented using stills of several time points (Figure 3A). At t=0 s recording started, several seconds after addition of PMs. The first captured image already showed that PMs entered the cells, indicated by the white arrow. Within 28 seconds the indicated PM entered the cytoplasmic compartment and moved toward the center of the cell (t=71 s and movie S2, scale bar: 10 µm). Internalized PMs were surrounded by green signal, indicated by the yellow arrow and in the close up square. Since CAAX-GFP is localized as an integral part of the plasma membrane we explain this by assuming that internalization of PMs occurs with concurrent internalization of the cellular membrane. This indicates that uptake could occur via an endocytic pathway 37. The short time interval imaging revealed highly dynamic and directional movement of PMs after uptake (Movie S3, scale bar: 10 µm). Directionality of intracellular movements could point to processing of PMs by cell components such as microtubules 38.

We therefore expressed Tubulin-YFP in PNT2C2 cells to image the microtubules inside PNT2C2 after PM uptake. Again, cells were incubated with PKH26-labeled PMs and spinning disk microscopy was used for imaging of microtubules. The dynamic and directional movements of internalized PKH26-labeled PMs (white arrow) localized at microtubules (Figure 3B and movie S4, scale bar: 10 µm). The linear displacement movement speeds through the cytoplasm reached intracellular velocities up to 1 μm/s (Figure 3 C and D). Interestingly, velocity showed periodic peaks over-time, indicating 'pause' steps during the process. Co-localization with microtubules and periodic movements already shown for early-endosomes generates a second indication of endocytic uptake of PMs 39.

### Analysis of endocytic uptake of polymersomes

For further analysis of the intracellular fate of PMs we used co-localization studies. With the use of specific proteins or makers that label early-endosomes (Rab4a-YFP) and lysosomes (Lysotracker red) we investigated the co-localization of endocytic bodies and intracellular PMs. Rab4a-YFP expression constructs were transiently transfected into PNT2C2 cells. Rab4a-YFP (Excitation 514/Emission 525) was combined with PKH26 labeled PMs and Lysotracker red (Excitation 550/Emission 590) was combined with PKH67 labeled PMs. In a pulse-chase experiment we incubated cells with PMs (1E11 PMs/mL) for 30 minutes, washed with PBS and refreshed with complete medium. Treated cells were fixed at 30, 60, 90, 120, 180, 240 and 360 minutes after PBS-wash. At least 10 cells per time point were imaged for co-localization analysis of PMs compared to marked endocytic pathway components (Figure 3E, typical examples at 30 and 300 minutes after PBS wash). 50% of PMs (PKH26) co-localized with early-endosomes (Rab4A-YFP) at 30 minutes, which declined to 10% within 5 hours. In contrast, co-localization between Lysotracker red (lysosomes) and PKH67 labeled PMs increased to 60% within 6 hours after treatment (Figure 3F). These results showed the transition of PMs throughout the maturation of early-endosomes to lysosomes in the endocytic pathway. In addition, spinning disk microscopy of PNT2C2 cells transiently expressing Rab4a-YFP (early-endosomes) showed clear merging of Rab4a-YFP labeled organelles containing PMs, indicating fusion of early-endosomes to late-endosomes/lysosomes (Movie S5, scale bar: 10 µm). We conclude that *in vitro* uptake of PMs is mediated through the endocytic pathway, where PMs enter the cell via early-endosomes and ultimately accumulate in lysosomes.

### High-resolution analysis of intracellular distribution of polymersomes

**Figure 4:**
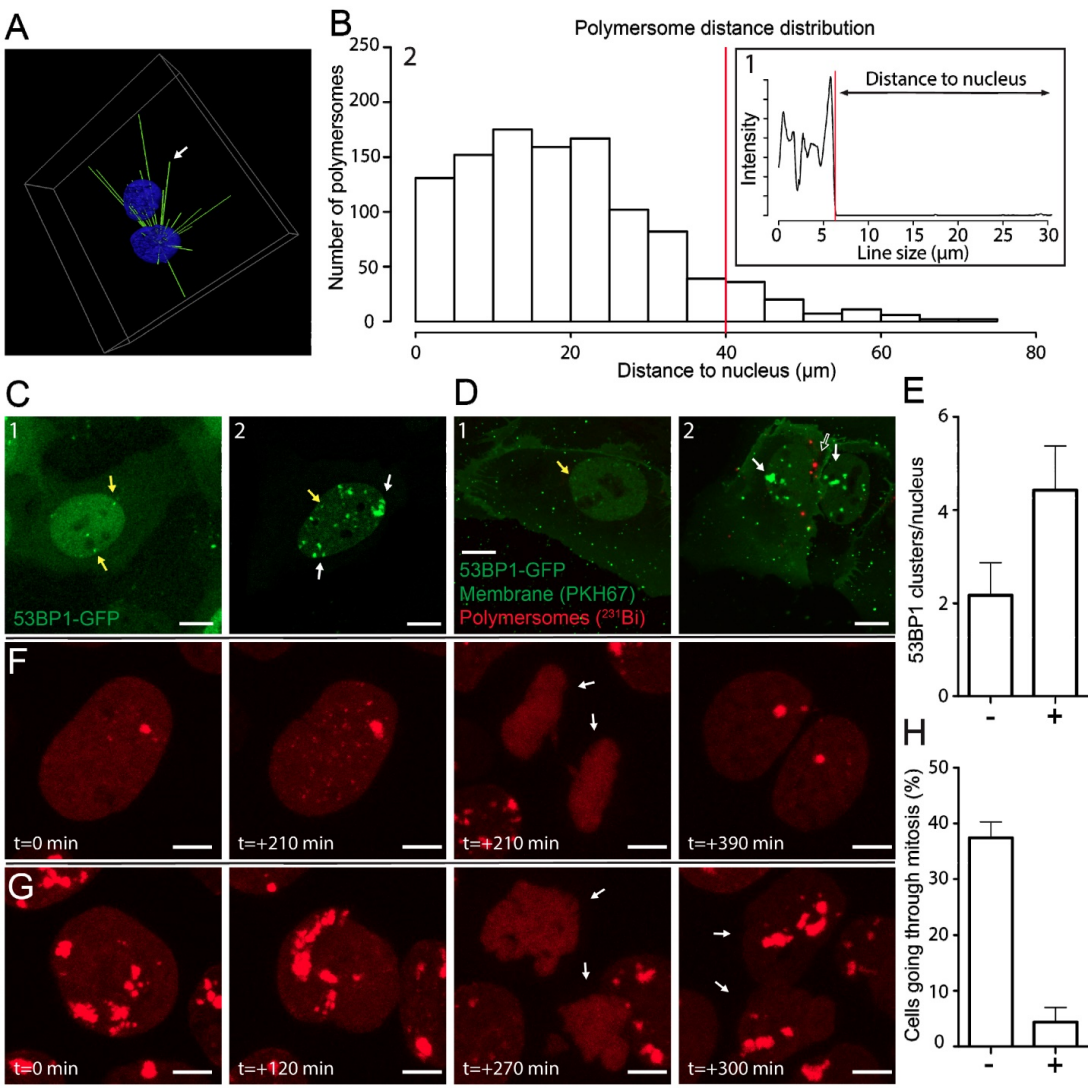
** Polymersome distance distribution of ^231^Bi labeled polymersomes and DNA damage induction.** PNT2C2 cells were incubated for 2 hours with 80 (±11) nm sized PKH26 labeled PMs. **(A)** 3D representation of the distance calculation between PM and the center of a nucleus. White arrow indicates the position of one PM. The intensity profile on the right shows the intensity of nuclear staining measured on that straight line. Distance of PM to nucleus is the determined as the line size between the clear drop of nucleus signal to PM position. **(B)** Example of an intensity profile measured on a straight line in between a PM and the center of a nucleus. Red line indicates the threshold for the edge of the nucleus (B.1). Overall distance distribution of PMs to nucleus (N=1091). Red line shows the relevant 40 µm distance cut off (B.2). **(C)** U2OS cells expressing 53BP1-GFP as DNA-damage marker. Typical examples of U2OS cells expressing 53BP1-GFP at control levels (C.1) and after alpha-particle irradiation (C.2). Yellow arrows indicate endogenous 53BP1-GFP foci and white arrows indicate DNA damage caused by alpha-particle irradiation. **(D)** U2OS cells expressing 53BP1-GFP were incubated with PMs (empty arrow) labeled with ^231^Bi (0.15 MBq) for 3 hours. Membrane was labeled with PKH67. Yellow arrows endogenous 53BP1-GFP foci (D1). White arrows indicate alpha-particle induced DNA damage. Empty arrow indicates radiolabeled PMs (D1). **(E)** DNA damage quantification. 7 cells without (-) or with (+) intracellular PMs (total = 14 cells) were evaluated for amount of 53BP1 foci. Error bars show SEM. Scale bar represents 10 µm. (F) U2OS cells expressing mScarlet-t53BP1 as DNA-damage marker. Typical examples of cells going through mitosis without DNA damage **(F)** and after alpha-particle irradiation (G). Arrows indicate the 2 daughter cells after mitosis. Time points indicate time in between snap shots. Scale bar represents 5 µm. (H) Quantification of mitosis. N= 38 for non-irradiated cells (-), N = 95 for irradiated cells (+) Error bars show SEM.

The position of PMs is highly dynamic during the processing of endosomes to lysosomes. The dynamic distribution of PMs throughout this process could greatly influence the efficiency of energy deposition of alpha particle irradiation 40. The information of the exact distance of PMs to nuclei at certain time points could be of great use, considering the short path-length of alpha particles. With the use of Structured Illumination Microscopy (SIM) we determined the position of intracellular PMs with high precision and evaluated per nucleus the number of PMs which were in range to deposit alpha particle radiation to the nucleus. We treated PNT2C2 cells with PKH26 labeled 80 nm PMs for 2 hours, washed with PBS, fixed, and stained the nuclei with DAPI. Using 3D analysis, we drew lines between centers of nuclei and PMs (Figure 4A and movie S6). By measuring the intensity profile of the nuclear staining on the straight lines we calculated the distance between the edge of the nucleus and all PMs in a single image. We use the drop of sirDNA signal as indication of the edge of the nucleus (Figure 4B.1). Distances between the edge of the nucleus to the PM coordinate was defined as 'distance to nucleus'. By calculating all PM to nucleus distances we generated a distance distribution (Figure 4B.2). The histogram shows that most PMs (1008 of 1091, 92%) are within the range of the supposed alpha particle path length (<40 μm, red line, median: 17.7 µm). We conclude that many PMs can contribute to effective alpha particle irradiation of cell nuclei in a 2-D setting. To test this, the efficacy with which DNA damage could be induced could be investigated using radiolabeled PMs with alpha particle emitting radionuclides.

### ^213^Bi labeled polymersomes damage nearby nuclei of U2OS cells after uptake

Positioning of PMs in the cytoplasmic compartment of cells could be crucial for the effective DNA damage induction when irradiation is used. We used the DNA damage marker p53 binding protein 1 (53BP1) fused to GFP to visualize DNA damage inflicted by radioactivity in U2OS cells 41. In addition, we used PKH26 and PKH67 to visualize PMs and the plasma membrane, respectively. Typical examples of 53BP1-GFP control conditions and after external alpha particle irradiation are used as reference to PM treated samples. A few small 53BP1-GFP clusters were present when no DNA damage is induced (yellow arrows, Figure 4C.1) compared to numerous and large 53BP1-GFP clusters after alpha particle induced DNA damage (white arrows, Figure 4C.2).

To investigate DNA damage inflicted by alpha particles, originated from radiolabeled PMs, we used ^213^Bi as radionuclide. Radioactive labeling of PMs showed efficiencies up to 49.2% for ^213^Bi and the activity measured was 372 kBq/mL. U2OS cells were treated with 2E12 ^213^Bi-PMs/ml (372 kBq) for 3 hours, washed with PBS and fixed. Confocal imaging was used to determine the spatial distribution of double strand breaks (53BP1-GFP clusters) and PMs after treatment (Figure 4D). Nuclei of cells with intracellular PMs (Figure 4D.2, unfilled arrow) showed increased 53BP1-GFP clusters, compared to nuclei of cells with no intracellular PMs (Figure 4E.1). Quantification showed a 2-fold increase of 53BP1-GFP foci in the nucleus if ^213^Bi labeled PMs are in the cytoplasmic compartment of U2OS cells (Figure 4E). These results are comparable with the external alpha particle irradiation experiments, indicating direct DNA damage induction by intracellular ^213^Bi labeled PMs but no apparent DNA damage induction to nuclei in close vicinity. To investigate the fate of alpha particle irradiated U2OS cells at later time points we used live-cell imaging. U2OS cells transiently transfected with mScarlet-t53BP1 (a truncated version of 53BP1) were externally irradiated with alpha particles and imaged immediately. Snapshots of both non-irradiated and irradiated cells show that both go through cell division (Figure 4F and G, movie S7). Interestingly, alpha particle induced DNA damage did not prevent cell division. However, quantification showed that cells not hit by alpha particles showed significantly more dividing cells than cells that did got hit (32% vs 3.2%, Figure 4H). We conclude that ^213^Bi-PMs induced DNA damage to U2OS nuclei when PMs were present in the cell, comparable with DNA damage when external alpha-irradiation was used. When PMs were not intracellular, no apparent induction of DNA damage was observed. Cells hit by alpha particles are possibly delayed or restricted to go through mitosis compared to cells that did not get hit. Moreover, our improved understanding of PM internalization assists in accurate prediction of nuclear DNA damage induction by radionuclide carrying PMs in targeted cells.

## Discussion

PMs have high potential in targeted alpha radionuclide therapy, while in addition the recoil problem of high-LET radionuclides could be solved. In this study we investigated the cellular uptake and intracellular processing of PMs to elucidate the uptake characteristics of PBd-PEO based PMs. Our work demonstrates that altering PM size and concentration affects the initial rate of uptake and overall uptake capacity. In addition, PM uptake varies between cell lines and cells undergoing mitosis have an increased PM uptake. High-speed live cell microscopy shows that PMs enter cells, co-localize with membrane components, and are transported along microtubules in a dynamic fashion. Evidence for endocytic uptake of PMs was obtained from co-localization of PMs and endocytic pathway compartments. Moreover, we show that the intracellular distribution and the DNA damage induced by ^213^Bi labeled PMs can best be described by a model that takes into account only damage caused by PMs in their cell of residence.

PM uptake and efficiency by macrophages (J774) was similar to prostate cancer cells (DU145) but less than osteosarcoma (U2OS) and prostate-epithelial (PNT2C2) cells (Figure 1B). Cell size or shape could have impact on the uptake rate and plateau levels of PM uptake. Larger cells have more surface area for PMs to enter and less membrane tension which leads to increased uptake efficiency 42. Unexpectedly, macrophages did not show disproportionately faster or increased uptake compared to cancer or epithelial cell lines. This is in contrast to several *in vivo* reports where high levels of liver and spleen uptake was observed. Uptake in these organs is explained by the presence of macrophages, for example Kupffer cells in the liver 19, 43, 44. A possible explanation for the difference in the data obtained *in vivo* versus the data from cultured cells could be the immortalization of J774 cells. Immortalized cell lines could acquire altered properties over time in cell culture 45. Including primary cells, for example harvesting Kupffer cells from the liver, could provide a more realistic setting 46. On the contrary, specific uptake by tumour-associated macrophages could induce polarization, which is thought to be beneficial for immune-therapies 47.

In addition to cell type or size, cell-cycle state could also have an impact on PM uptake. Live-cell imaging shows increased PM uptake at the point of mitosis. The alteration of the cell membrane during mitosis could allow PMs to enter the cells 48. In addition, the change of cell shape during mitosis is accompanied by recycling membrane components to intracellular compartments 49. The increased uptake of PMs by dividing cells suggests enhanced uptake in frequently dividing tumor cells, important for therapeutic approaches using PMs 50. The uptake of PMs we observe in our experiments and encounter in previous literature suggests that endocytosis is the most probable pathway by which PMs enter cells 51. Co-localization experiments confirmed that PMs enter the cell and reside in early-endosomes, eventually ending up in lysosomal compartments. In addition, intracellular processing of PMs shows co-localization with microtubules and bi-directional movement patterns and speeds of 0.2 to 1 μm/s for 4 measured PMs. The measured velocities are comparable with previously reported values of 0.6 μm/s 52, 53. Since the PMs we employed are non-biodegradable we assumed that they remain in the cytoplasm for longer periods than we have investigated and form bulky lysosomal compartments 54. While we show the co-localization with endocytic compartments during PMs entry and processing, the exact endocytic pathway remains to be elucidated. The use of haploid-screening could be used to determine important genes for PMs uptake, shown in similar experiments for viral entry and chemotherapeutic sensitization 55, 56.

Aside from PM uptake, knowledge of intracellular localization is desired for (micro)dosimetry calculations. Simulations have shown that radionuclides in close vicinity of the nucleus have higher RBE compared to radionuclides at the membrane 40. Theoretically, the amount of DNA damage is dependent on the position of the Bragg peak 57. Radiolabeled PMs localized close to the nucleus should therefore have a higher chance to induce DNA damage due to geometric considerations. Peri-nuclear localization and lysosomal aggregation after uptake of non-biodegradable nano-carriers is suggested and described in literature 58. This was observed in U2OS cells already 1 hour after PM addition, but not in PNT2C2 cells, which show a more random distribution (Figure 1B). Our data suggest a random distribution of multiple PMs within 40 µm of surrounding nuclei in a 2-D setting in PNT2C2 cells (Figure 4B). In addition, PMs are being trafficked throughout the cytoplasm via microtubules in the investigated time span and eventually accumulate in the lysosomes (Figures 3B and E, Movies S2 and S3). This movement and positioning is dependent on time, but also on cell type (Figure 1B, 59). The continuous movement makes a direct correlation of PM location and the induction of DNA damage very challenging. In addition, recent work highlights the need for active targeting of PMs, especially in the presence of a tumour 60. This could possibly change the uptake and processing dynamics of PMs, both *in vitro* and *in vivo* 61-63.

However, the analysis presented here could function as a prerequisite for the interpretation of 2-D experiments for comparison to 3-D *in vitro* systems, where radiation from neighbouring cells is much higher 21. In addition, in current experimental set-up, the small amount of PMs translates to a small amount of alpha particles that are being emitted and a low delivered dose to cells. Using ^213^Bi, we provided preliminary insight of possible therapeutic benefit. However, the singular alpha emission of ^213^Bi, does not provide enough information of the benefit of intracellular PMs. ^225^Ac, for example, has multiple alpha emissions in its decay chain. The use of ^225^Ac has seen therapeutic potential in glioma models 21. For future studies, the use of ^225^Ac might be more relevant to investigate the benefit of geographically fixing radiolabeled PMs intracellularly. Moreover, our observations suggest that in a 2-D setting, most of the alpha particles go out of plane, not hitting a nucleus. This makes it hard to explain an one-to-one ratio correlation of DNA damage with the intracellular PMs responsible of emission. This demonstrates the impact of localization and uptake efficiency of PMs labeled with alpha particle emitting radionuclides.

In conclusion, PM uptake is mediated via endocytosis and is particle size, concentration, and cell type dependent. Our findings suggest that PM and, in theory, other nano-carrier uptake and processing can differ significantly between cell lines. This difference will influence the effective DNA damage induction by the radionuclides encapsulated by PMs*.* Analysing uptake characteristics using the assays presented in this study will help to provide crucial information, such as DNA damage inducing capabilities and effective uptake. In addition, the assays provide techniques to study the effect of specific characteristics of nano-carriers, for later therapeutic use. Future experiments with PMs and other nano-carriers will benefit from the advanced analysis presented here and results should be considered for increased efficiency of optimization for new nano-carriers.

## Supplementary Material

Movie S1: Dividing cells.

Movie S2: polymersome entering.

Movie S3: Dynamic polymerome processing.

Movie S4: Microtubule colocalization.

Movie S5: Endosomal merging.

Movie S6: 3D analysis of polymersome distribution.

Movie S7: Live cell imaging of 53BP1.

## Abbreviations

TAT: targeted alpha therapy
LET: linear energy transfer
RBE: relative biological effect
PM: polymersome
EPR: enhanced permeability effect
PEG: polyethylene glycol
SIM: structured illumination microscopy
PBd: polybutadiene
PEO: polyethylene oxide
53BP1: p53 binding protein 1
